# Pathology of the outbreak of subgenotype 2.5 classical swine fever virus in northern Vietnam

**DOI:** 10.1002/vms3.339

**Published:** 2020-08-11

**Authors:** Uda Zahli Izzati, Nguyen Thi Hoa, Nguyen Thi Lan, Nguyen Van Diep, Naoyuki Fuke, Takuya Hirai, Ryoji Yamaguchi

**Affiliations:** ^1^ Department of Veterinary Pathology Faculty of Agriculture University of Miyazaki Miyazaki Japan; ^2^ Faculty of Veterinary Medicine Vietnam National University of Agriculture Gia Lam Hanoi Vietnam

**Keywords:** genotype 2, hog cholera, moderately virulent, natural infection, pathology

## Abstract

Classical swine fever (CSF) is an endemic disease in southeastern Asia and is one of the most important swine diseases in Vietnam. This study was conducted to characterize the pathology of natural cases of CSF in northern Vietnam in 2018 and their genetic prevalence. A total of 10 representative pigs were collected from four provinces (Hung Yen, Ha Noi, Quang Ninh and Thai Binh) during five outbreaks and examined pathologically. The gross and histopathological findings showed the disease was expressed as the acute or the subacute to chronic form of CSF, depending on the age of the animals. The most consistently observed lesions associated with infection by the classical swine fever virus (CSFV) included lymphoid depletions in tonsils, lymph node and spleen; histiocytic hyperplasia in spleen; cerebral haemorrhage; perivascular cuffing in the brain; renal erythrodiapedesis; urothelial vacuolation and degeneration and interstitial pneumonia. The immunohistochemical findings showed a ubiquitous CSFV antigen mainly in the monocytes/macrophages and in the epithelial and endothelial cells in various organs. CSFV neurotropism was also found in the small neurons of the cerebrum and the ganglia of the myenteric plexus. Analysis of the full‐length envelope protein (E2) genome sequence showed that all strains were genetically clustered into subgenotype 2.5, sharing a nucleotide identity of 94.0%–100.00%. Based on the results of this study, the strain was categorized as a moderately virulent CSFV.

## Graphical abstract

In this study, natural cases of classical swine fever in northern Vietnam showed variable degree of lymphoid depletions in the lymphoid organs including lymph node, tonsil and spleen; with the remaining parenchyma replaced with reticulo‐histiocytic hyperplasia. This figure showed the sinus haemorrhage in the mesenteric lymph node and CSFV antigen was detected in the mononuclear cells by IHC.

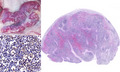

## 1 INTRODUCTION

The causative agent of classical swine fever (CSF) is the classical swine fever virus (CSFV), or Pestivirus C, which belongs to the *Pestivirus* genus within the *Flaviviridae* family (Smith et al., 2017). CSFV is highly contagious in porcine species, especially domestic pigs and wild boars; however, a recent study has reported isolation of the virus from bovine species (Kirkland et al., 2012; Valli, Kiupel, Bienzle, & Wood, 2016; Giangaspero, Kumar, & Zhang, 2017). The clinical form of CSF can be categorized as acute (transient or lethal), chronic and pre‐natal (late onset) but, historically, CSF may also occur as acute, subacute, chronic, atypical or inapparent syndromes, depending on the virus strain and host immunity (Moennig, Floegel‐Niesmann, & Greiser‐Wilke, 2003; Valli et al., 2016). Virulent strains of CSFV cause acute CSF with high morbidity and mortality, whereas moderately virulent strains may cause subacute to chronic CSF and low‐virulence strains may cause asymptomatic disease (Valli et al., 2016).

The most common CSF lesions in acute cases are renal petechiae, splenic infarction (incidence varies from 1% to 87% depending on the strain of virus), haemorrhages in the periphery of lymph nodes, perivascular cuffing in the brain (formed by transmural migration of monocytes), vascular lesions (lesions vary from a slight thickening of the capillary wall to fibrinoid necrosis of arterioles and vasculitis) and lesions in the lymph nodes (these vary from slight oedema and proliferation of the reticuloendothelial elements to extensive haemorrhage and necrosis) (Robinson & Robinson, 2016). Subacute to chronic cases are most commonly characterized by button ulcers on the colonic mucosa (Robinson & Robinson, 2016), whereas the surviving piglets with pre‐natal CSF may be runts and may die within 2–12 months after birth from lesions that include thymic atrophy, pale swollen lymph nodes and focal colonic mucosal necrosis (Robinson & Robinson, 2016). Until recently, CSFV was divided into three genotypes with three or four subgenotypes in each group (Blome, Staubach, Henke, Carlson, & Beer, 2017). A recent study proposed a new CSFV classification consisting of five main genotypes (1–5), with seven subgenotypes in each of genotype 1 and 2 (Rios, Núñez, Díaz de Arce, Ganges, & Pérez, 2018). At present, no clear correlation has been established between the CSFV genotype and virulence, but the most virulent strains are found in genotype 1, whereas the strains in genotype 2 tend to be moderately virulent (Beer, Goller, Staubach, & Blome, 2015).

CSF is endemic in swine in Vietnam, and the CSFV strains recently reported in northern Vietnam belong to genotype 2 (Hung, Lan, Nga, & Truong, 2017; Kim et al., 2019). Animal husbandry in Vietnam tends to be performed by many small holders whose farming practices have many deficiencies (Ngan et al., 2016) that can potentially aggravate the progression of diseases like CSF and the evolution of its aetiological agents. Therefore, characterization of the pathology of CSF cases and their epidemiological status is necessary to understand the current situation of the disease and to develop control countermeasures. The aim of this study was to obtain a clear picture of the histopathological changes occurring in natural cases of CSF in swine in the endemic region and to obtain genetic information about the virus.

## 2 MATERIALS AND METHODS

### 2.1 Sample collection

Routine necropsies were conducted at the Vietnam National University of Agriculture (VNUA) on diseased pigs that were collected from farms in four provinces in northern Vietnam during disease outbreaks in March and April 2018. The total number of outbreaks was 5 (outbreak 1: Pigs 1–5; outbreak 2: Pig 6; outbreak 3: Pig 7; outbreak 4: Pig 8; outbreak 5: Pigs 9–10). The information about the pigs used in this study is summarized in Table 1. Fresh tonsil and spleen tissues were collected from the pigs from these suspected CSF outbreaks and stored at −80°C until processed. Tissues collected from tonsil, brain, heart, spleen, stomach, intestines, urinary bladder, liver and lymph nodes were placed in 10% neutral‐buffered formalin solution and processed routinely.

**TABLE 1:** List of the classical swine fever outbreaks in northern Vietnam (2018) and CSFV isolates

District	Province	Mortality rate (%)	Vaccination	Pig number	Age (day)	Ct value qRT‐PCR	Isolate name	Accession number
Van Giang	Hung Yen	50–53	PRRS, Enzootic pneumonia, PCV2	1	20	18.34	HY20181	MK782036
				2	19	15.80	HY20183	MK782037
				3	20	19.27	HY20184	MK782038
				4	20	17.53	HY20186	MK782039
				5	15	18.21	HY20187	MK782040
Gia Lam	Ha Noi	‐	‐	6	—	16.79	HN20182	MK782041
				7	—	17.43	HN20183	MK782042
Dong Trieu	Quang Ninh	10	PRRS, MH, CSF	8	50	16.63	QN201810	MK782043
Dong Hung	Thai Binh	<1	CSF, PRRS, circovirus, FMD, Aujezsky's disease	9	>180	21.27	TB20181	MK782044
				10	>180	18.34	TB20182	MK782045

Note

—: No information

Abbreviations: PRRS, Porcine reproductive and respiratory syndrome; PCV2, Porcine circovirus type 2; MH, *Mycoplasma hyopneumoniae*; CSF, Classical swine fever; FMD, Foot and mouth disease.

*Ethics statement:* Diseased pigs were collected at the moribund stage and killed by veterinarians. All sampling procedures were conducted during necropsy; thus, no unnecessary pain was inflicted on the animals.

### 2.2 CSFV detection and screening

Pig necropsies conducted at VNUA were screened for CSFV using a real‐time one‐step reverse transcriptase‐polymerase chain reaction (qRT‐PCR) with a Taqman probe. RNA was extracted from the tonsil and spleen tissue using the PureLink™ RNA Mini Kit (Invitrogen). The Superscript III Platinum Onestep qRT‐PCR (Invitrogen) kit was used for the qRT‐PCR with the following primers: forward primer: ATG CCC WTA GTA GGA CTA GCA; reverse primer: TCA ACT CCA TGT GCC ATG TAC; and Taqman probe: TGA TGG GGG TAC GAC CTG ATA GGG T (Hoffmann, Beer, Schelp, Schirrmeier, & Depner, 2005; McGoldrick et al., 1998). A 40 µM concentration of each primer and 10 µM of the probe were used in the reaction. The samples were considered positive when the cycle threshold (Ct) value was <35, suspected positive when the Ct value was >35 and deemed negative when no Ct value was obtained. Upon confirmation of CSFV, complementary DNA was produced from the RNA extract using the GoScript Reverse Transcription System (Promega) and subjected to sequence analysis. Other viral pathogens, including porcine circovirus type 2 (PVC2), porcine reproductive and respiratory syndrome virus, swine influenza virus and porcine cytomegalovirus (PCMV), as well as bacterial pathogens, including *Salmonella spp., Streptococcus suis*, *Actinobacillus pleuropneumonia* and *Haemophilus parasuis,* were detected using the primers listed in Supplementary data 1. The paraffin‐embedded, formalin‐fixed tissue samples were examined for histopathological evaluation.

### 2.3 Histopathological evaluation

The histopathological evaluation was conducted on 4 µm thin sections of paraffin‐embedded formalin‐fixed tissue samples stained with haematoxylin/eosin (HE). All lesions observed in the HE staining tissue specimens were examined.

### 2.4 Immunohistochemical (IHC) detection of CSFV antigen

The immunohistopathological evaluation used 4 µm thin sections of the paraffin‐embedded formalin‐fixed tissue samples. The WH303 monoclonal antibody (APHA Scientific) specific for the E2 (gp53) glycoprotein of CSFV was used as the primary antibody. Following heat‐induced antigen retrieval in citrate buffer (pH6.0), the WH303 antibody was diluted at 1:100 according to the manufacturer's recommendation in a Blocking One (Nacalai Tesque) solution and incubated with the tissue specimen at 4°C overnight in a humidified chamber. Histofine® MAX PO Multi (Nichirei Biosciences) was used as the secondary antibody. The reaction was visualized using 3,3'‐diaminobenzidine (Sigma‐Aldrich) chromogen. Tissue specimens drawn from a pig at a CSFV‐free farm were used as the negative control.

### 2.5 Full E2 sequence and phylogenetic analysis

The CSF2250f, CSF2919f, CSF3010f, CSF3000r and CSF3710r primers described previously were used for the full‐length sequence of E2 glycoprotein genome (Postel et al., 2012). The amplified PCR products were purified using a FastGene Gel/PCR Extraction kit (Nippon Genetics). The purified amplicons were subjected to a double‐stranded Sanger sequencer. The sequence data were analysed and edited using the BioEdit Sequence Alignment Editor (Hall, 1999). A total of 10 sequences were submitted to the GenBank databases under accession number MK782036 to MK782045. The accession numbers of each pig are listed in Table 1. Forty‐eight full‐length E2 sequences were retrieved from the gene bank following the new classification proposed for phylogenetic analysis (Rios et al., 2018). The following E2 gene sequences of CSFV strains with recognized virulence were retrieved from the gene bank or the Classical Swine Fever Database (EU and OIE Reference Laboratory for CSF) and included in the phylogenetic analysis: high virulence Alfort/187 (Gómez‐Villamandos et al., 2006), ALD (Narita et al., 2000), Brescia, Eystrup (Floegel‐Niesmann, Bunzenthal, Fischer, & Moennig, 2003), CSF1047 (Lohse, Nielsen, & Uttenthal, 2012); moderate virulence CSF0277 and CSF0634 (Floegel‐Niesmann et al., 2003), CSFV/Mongolia/Bu08/2014 (Enkhbold et al., 2017), 94.4/IL/94/TWN (Lin, Chien, Deng, & Huang, 2007); and low‐virulence Kanagawa 74 (Narita et al., 2000). The CSFV sequence derived from the reported HY78 strain in Vietnam in 2015 and the CSFV/JPN/1/2018 strain in recent outbreaks in Japan were also included (Kim et al., 2019; Nishi, Kameyama, Kato, & Fukai, 2019). The *Pestivirus* Aydin strain was used as an outgroup. The accession numbers of all 66 sequences used in this study are listed in Supplementary data 2. The phylogenetic analysis, which was based on the CSFV classification proposed in 2018 (Rios et al., 2018), was conducted by the maximum likelihood (ML) approach with 1,000 bootstrap replications using MEGAX software (Kimura, 1980; Kumar, Stecher, Li, Knyaz, & Tamura, 2018). The sequence identity matrix was plotted using the BioEdit Sequence Alignment Editor to calculate the nucleotide identity (Hall, 1999).

## 3 RESULTS

### 3.1 Detection of CSFV by qRT‐PCR

The Ct values detected in all cases are tabulated in Table 1. The PCR results of various viral and bacterial pathogens are included in the Supplementary data 3. RT‐PCR or PCR of other viral and bacterial pathogens yielded negative results, except that PCV2 and PCMV were detected in several cases.

### 3.2 Gross and histopathological findings

**FIGURE 1:**
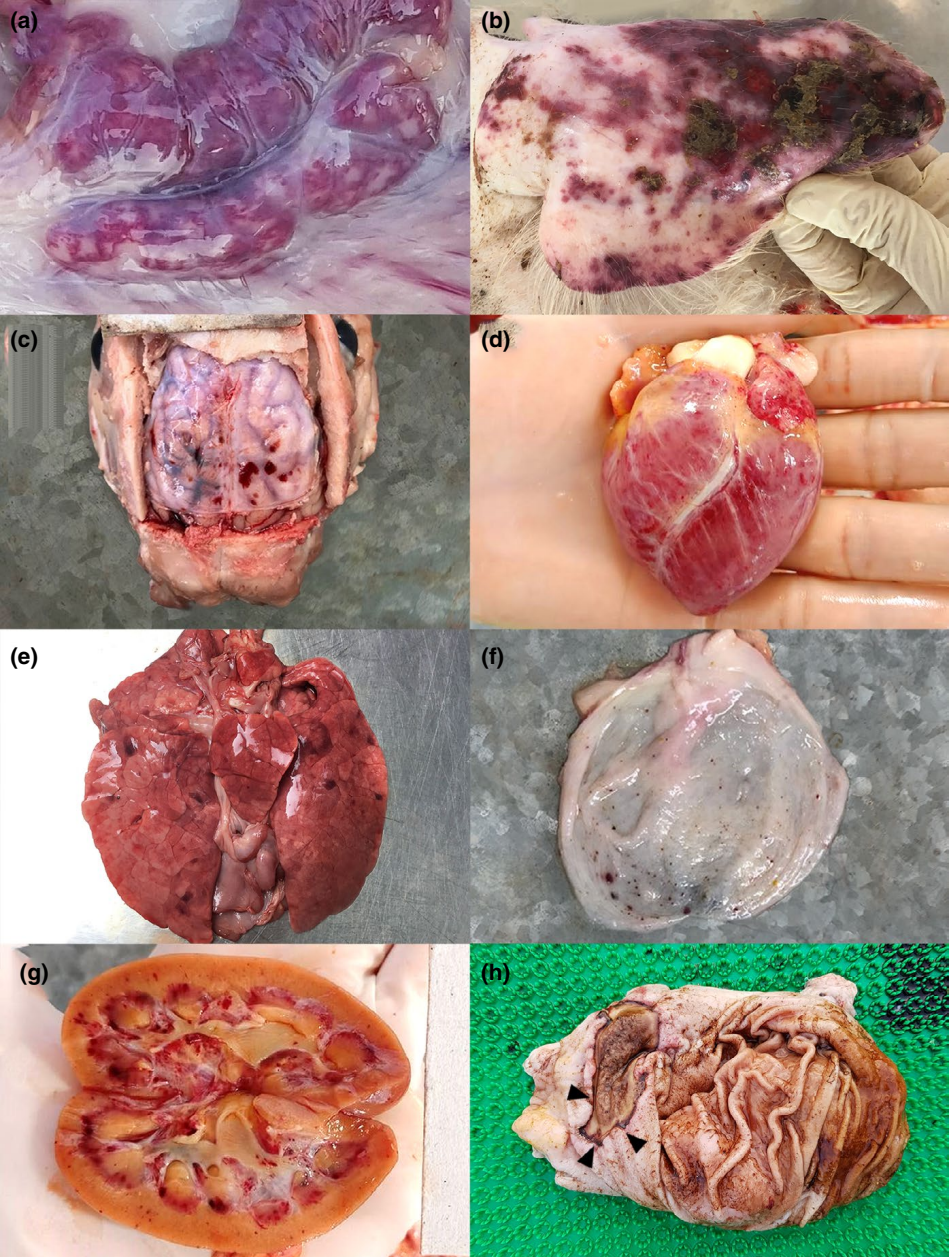
Gross image of classical swine fever infection in pigs (a) Sinus haemorrhages in mesenteric lymph node of Pig 10; (b) Erythema and hyperaemia in ear lobe in Pig 6; (c) Meningeal haemorrhages in Pig 5; (d) Pericardial haemorrhages in Pig 2; (e) Ecchymotic pleural haemorrhages in Pig 4; (f) Petechial haemorrhages on urinary bladder mucosa in Pig 5; (g) Petechial haemorrhages in renal cortical and medulla in Pig 2; (h) Gastric ulcer in Pig 9 (arrowhead)

Macroscopically, the CSF lesions included sinus haemorrhage in the lymph nodes (7/10) (Figure 1a), erythematous skin (6/10) (Figure 1b), cerebellar or cerebral haemorrhage (3/6) (Figure 1c), petechial haemorrhage in the larynx (4/10), ecchymotic haemorrhage in the epicardium (3/10) (Figure 1d) and lung (4/10) (Figure 1e) and petechial haemorrhage in the urinary bladder mucosa (4/10) (Figure 1f) and kidney (5/10) (Figure 1g). A few animals showed necrotizing ulcers in the stomach (4/10) (Figure 1h), petechial haemorrhage in the small intestinal mucosa (2/10) and button ulcers in the colon (3/10). The gross changes are tabulated in Supplementary data 4.

**FIGURE 2:**
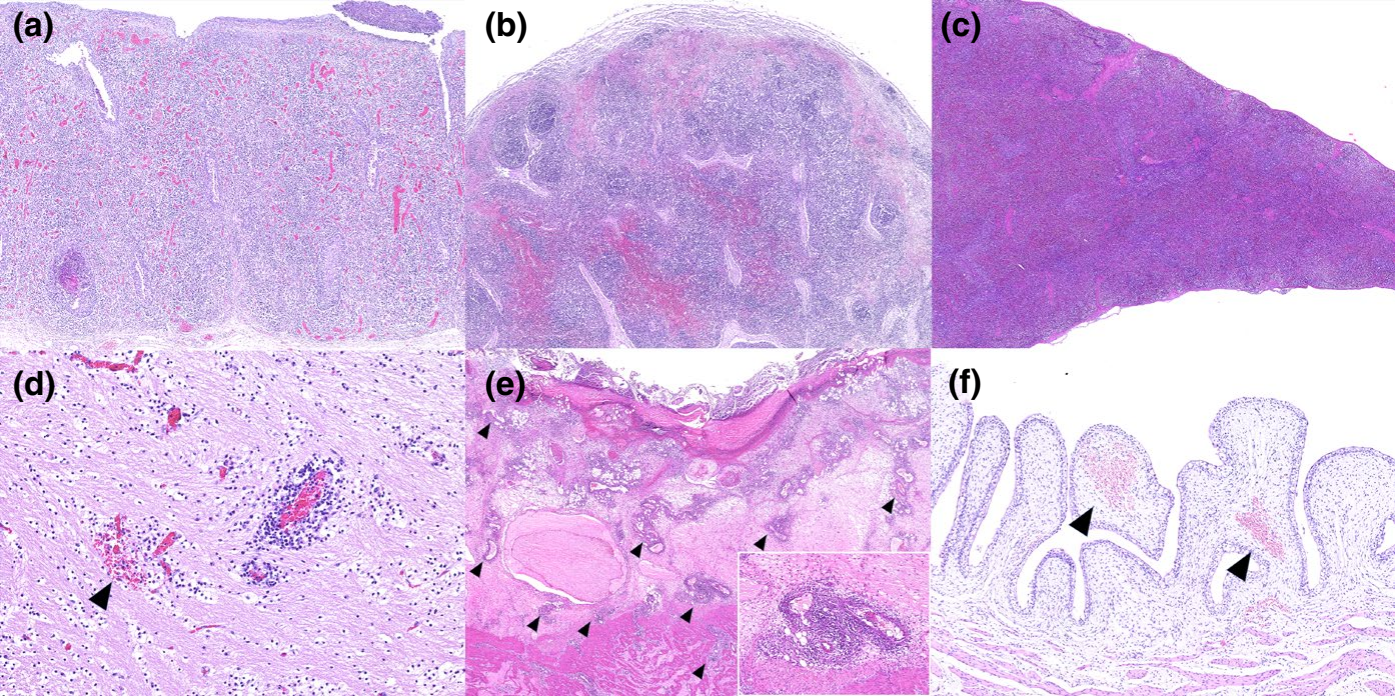
Histopathological changes seen in classical swine fever, HE staining. (a) Tonsil, Pig 1. Lymphoid depletion in tonsil with congestion. A small dilated crypt filled with necrotic cell debris can be seen in the lower left area; (b) Lymph node, Pig 4. Sinuses in the medullary lymph node are filled with erythrocytes and lymphoid follicles are depleted; (c) Spleen, Pig 2. A few small lymphoid follicles are present in the spleen. The red pulp is replaced by reticulo‐histiocytic proliferation; (d) Cerebrum, Pig 1. Perivascular cuffing is seen in capillaries in cerebrum. Microhaemorrhage is present in vicinity of capillary (arrowhead); (e) Stomach, Pig 9. Transmural necrotizing tissue in the gastric ulcer lesion. Vasculitis and/or perivasculitis are seen in almost all of capillaries present here (arrowheads, inset); (f) Urinary bladder, Pig 2. Urothelium show mild vacuolations. Submucosal haemorrhages are present (arrowheads), but there is no vasculitis

The histopathological lesions are summarized in Table 2. When viewed with a microscope, the most consistent lesions in all cases showed lymphoid depletion and histiocytic hyperplasia in the lymphoid organs, including tonsil, lymph node and spleen (Figure 2a–c). Some cases showed a severe degree of sinus haemorrhage in areas of necrosis (5/10). In the spleen, lymphoid depletion was usually less severe than in the lymph nodes, but histiocytic hyperplasia was observed in this study (8/8) (Figure 2c). Extramedullary haematopoiesis was observed in the spleen of Pig 10. Vasculitis and perivasculitis were most prominent in the brain (3/4) (Figure 2d), but were only occasionally detected in the heart (1/10), in ulcerated areas of the stomach (2/10) (Figure 2e) and in the colonic button ulcers (2/10). Lesions in the brain consisted of microhaemorrhages adjacent to blood vessels (2/4) and glial nodules (2/4). The urinary system commonly showed haemorrhages or erythrodiapedesis in the kidney (8/10) and submucosa of the urinary bladder (6/8) (Figure 2f). Most animals showed erythrodiapedesis in the renal cortical and medullary regions, but a few cases showed haemorrhages restricted to the cortical regions. Interstitial nephritis was seen in some cases (6/10), and a few cases showed focal renal necrosis (2/10). Urothelial vacuolation (9/9) and total exfoliation of the urothelial cells were sometimes observed in the renal pelvis and mucosal surface of the urinary bladder. A single case (Pig 8) showed focal perivasculitis in the urinary bladder submucosa.

**TABLE 2:** Histopathological attributes to natural cases of classical swine fever in northern Vietnam (2018)^a^

Organ	Lesions	Acute to subacute cases					Subacute to chronic cases				
		Pig number									
		1	2	3	4	5	6	7	8	9	10
Lymphoreticular organ	Splenic histiocytic hyperplasia	++	+++	++		++		+	+	+++	+++
	Lymphoid depletion	+++	+++	+++	+++	++	+++	+	+++	+++	++
	Lymph node necrosis	−	−	−	−	+	+++	++	+++	−	++
	Sinus congestion/ haemorrhage (lymph node)	+++	+++	−	+++	+++	−	+++	+++	−	+++
Gastrointestinal tract	Necrotizing ulcer (gastric)	−	−	−	−	−	++		−	+++^‡^	+++^‡^
	Submucosal haemorrhage (gastric)	−	−	−	−	++	−		−	−	+++
	Colonic button ulcer		−		−	+++^‡^	++		−	−	+++^‡^
Lung	Interstitial pneumonia	++	++	++	++	−	+	−	+++	+++	+++
	Bronchointerstitial pneumonia	−	+++	−	++	+	−	−	−	−	+++
	Alveolar haemorrhage	+	−	+	−	−	−	+	−	−	−
Kidney and urinary bladder	Renal erythrodiapedesis/ haemorrhage	+++	+	+	+	−	+	+	+++	−	+++
	Interstitial nephritis	−	+	+	−	+	−	−	+++	+++	+++
	Renal corticonecrosis	−	−	−	−	−	−	+	++	−	−
	Urothelial degeneration	+++	++	++	+++	+++	+		+	+	
	Urinary submucosal microhaemorrhage	+	+	+	−	+	−		+	+	
	Perivasculitis (urinary bladder submucosa)	−	−	−	−	−	−		+	−	
Heart	Subendocardial or subepicardial haemorrhage	−	−	−	−	+	−	+	++^b^	−	−
Brain	Perivascular cuffing	+++	−		++		+				
	Cerebral or cerebellar haemorrhage	+	−		+++		−				
	Glial nodule	+	+		−		−				

^a^ +: mild; ++: moderate; +++: severe; grey box: not done.

^b^ with vasculitis.

Interstitial pneumonia was commonly seen in the lung (8/10), and some animals showed bronchointerstitial pneumonia with variable severity (4/10). Lesions in the liver indicated mild to moderate pericholangiohepatitis (7/9), and a few animals showed micronecrosis in the periportal region (3/9). *Balantidium* sp. infection was seen in the necrotizing gastroenteritis in two cases (Pigs 5 and 6). Pig 10 showed severe necrotizing gastritis and ulcerative colitis without involvement of the *Balantidium* sp. organism.

### 3.3 Immunohistochemical evaluation of CSFV antigen distribution in tissue samples

**FIGURE 3:**
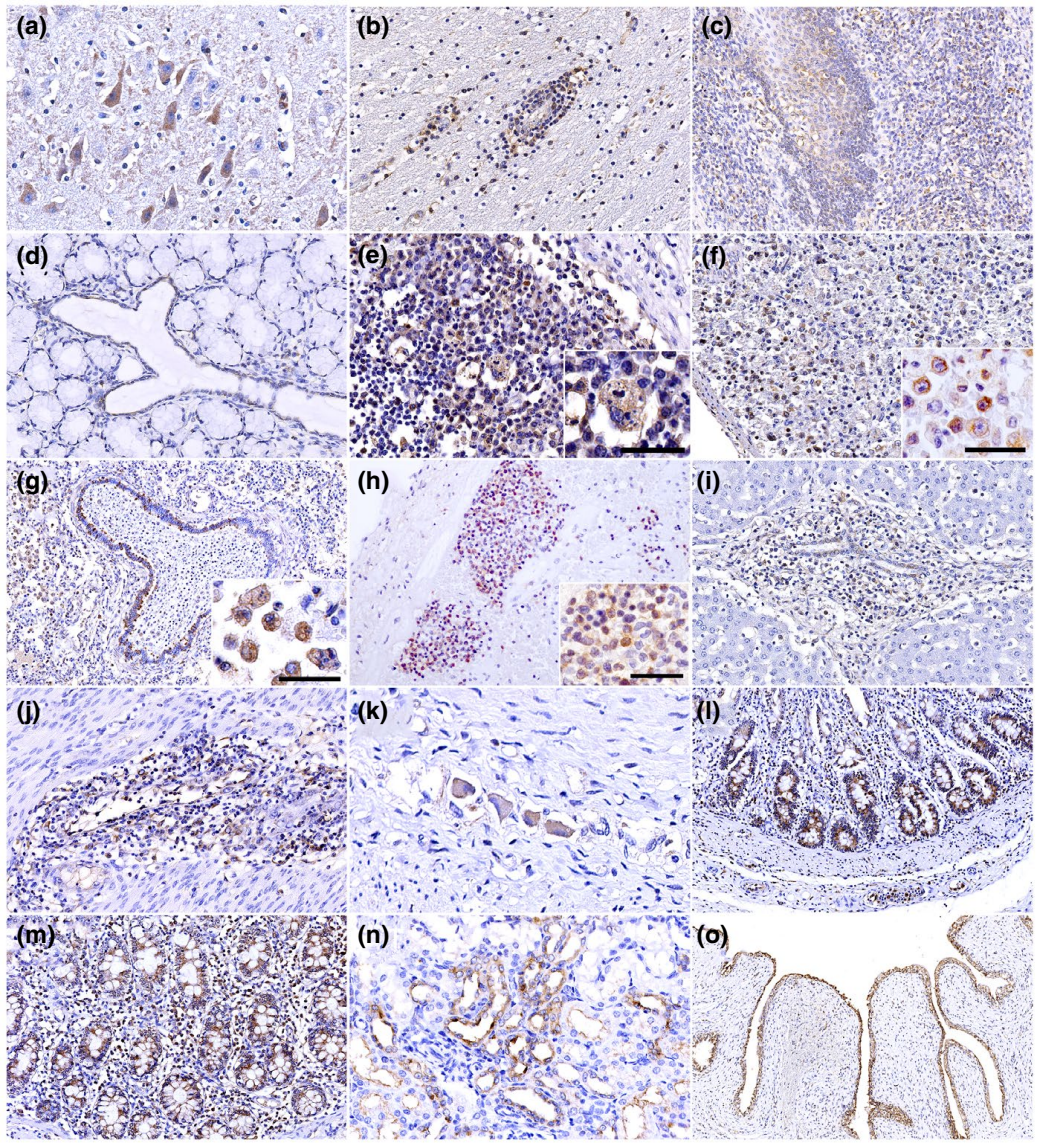
IHC for CSFV antigen detection. (a) Cerebrum, Pig 1. Small neurons in cerebrum are positive; (b) Cerebrum, Pig 1. Mononuclear cell in perivascular cuffing are positive; (c) Tonsil, Pig 10. Tonsillar epithelial crypt and surrounding mononuclear cells are positive; (d) Salivary duct, Pig 10. Salivary duct epithelium is positive; (e) Lymph node, Pig 4. Mononuclear cells are positive. Inset shows a macrophage with apoptotic body, bar: 20 µm; (f) Spleen, Pig 2. Mononuclear cells are positive, inset shows high magnification of positive cells, bar: 30 µm; (g) Lung, Pig 5. Macrophages populations in lung tissue are positive and bronchiolar epithelium showed multifocal‐positive staining, inset shows alveolar macrophages, bar: 30 µm; (h) Heart, Pig 8. Mononuclear cells in subendocardial haemorrhage area are positive. Myocardium is negative. Inset shows higher magnification of positive cells, bar: 30 µm; (i) Liver, Pig 5. Biliary ductal epithelium is mildly positive. Mononuclear cells surrounding portal duct are also positive; (j) Stomach, Pig 9. Endothelial cells in the necrotizing lesion are positive; (k) Stomach, Pig 1. Gastric plexus in the muscular layer show mild to moderate positive staining; (l) Small intestine, Pig 8. Strong positive staining in the villus epithelium; (m) Large intestine, Pig 9. Villus epithelium and the mononuclear cells within the lamina propria show positive staining; (n) Kidney, Pig 3. Renal tubular epithelium is positive; (o) Urinary bladder, Pig 3. Urothelium is homogenously positive

The CSFV antigen was ubiquitous in many organs, including the brain, tonsils, salivary duct, lymph nodes, spleen, lung, heart, liver, stomach, small and large intestine, kidney and urinary bladder (Figure 3a–o). The distributions of CSFV antigen‐positive cells and tissue components are summarized in Table 3. Monocyte‐macrophage components were primarily positive and abundant in the lymphoid organs (Figure 3c,e,f) and the capillaries in various organs (Figure 3l). In the lung, in addition to the pulmonary intravascular macrophages and interstitial macrophages, the alveolar macrophage population also showed positive staining (Figure 3g, inset). Monocyte‐macrophages in the lamina propria of the gastrointestinal tract were frequently positive (Figure 3l,m). Endothelial cells were positive in many organs, but they were distributed sparsely and were not necessarily associated with vasculitis and haemorrhagic areas. In two cases of necrotizing gastroenteritis, the endothelium was strongly positive within the vasculitis and perivasculitis lesions (Pigs 9 and 10). The endothelial cells within the perivascular cuffing in the brain were consistently positive in all cases observed (Pigs 1, 4 and 6). Epithelial components, including the tonsillar crypt epithelium, salivary duct, bronchiolar epithelium, renal tubules, urothelium and gastrointestinal tract epithelium, were consistently positive in 90% of the cases at varying intensities and mostly without apparent histological lesions, except for the vacuolation and exfoliation of the urothelium in the urinary bladder.

**TABLE 3:** Immunohistochemical detection of CSFV antigen in natural cases of classical swine fever in northern Vietnam (2018)^a^

	Monocyte‐macrophage	Epithelium	Endothelium	Neuron/Ganglia
Brain	3/4	na	^b^4/4	2/4
Tonsil	8/8	7/8	nd	na
Salivary duct	nd	6/7	nd	na
Lymph node	8/10	na	nd	na
Spleen	7/8	na	nd	na
Lung	10/10	10/10	7/10	na
Heart	5/10	na	5/10	na
Liver	6/10	2/10	0/10	na
Stomach	7/9	8/9	^c^7/9	5/9
Small intestine	6/8	8/8	3/8	3/8
Large intestine	4/6	5/6	^d^4/6	1/6
Kidney	1/10	10/10	2/10	na
Urinary bladder	6/8	8/8	5/8	na

^a^ Value shows the number of pig showing positive signal/total number of pigs observed; na, not applicable; nd, not done.

^b^ Antigen detection correlates with perivascular cuffing lesion in three cases.

^c^ Antigen detection correlates with vasculitis lesion in two cases (Pig 9 and 10).

^d^ Antigen detection correlates with vasculitis lesion in two cases (Pig 5 and 10).

The intensity of IHC staining was homogenous and the strongest in the urothelium, followed by the bronchiolar epithelium and gastrointestinal mucosal epithelium, whereas milder staining was observed in the tonsillar crypt epithelium and renal tubules; however, these observations were not accompanied by clear histopathological changes. Infrequently, small neurons in the brain (Pigs 1 and 2) and ganglia in the myenteric plexus were positive (Pigs 1, 2, 3, 5 and 9). All negative tissue controls (brain, tonsil, salivary duct, lymph node, spleen, lung, heart, liver, stomach, small and large intestine, kidney and urinary bladder) showed no staining.

### 3.4 Phylogenetic analysis

**FIGURE 4:**
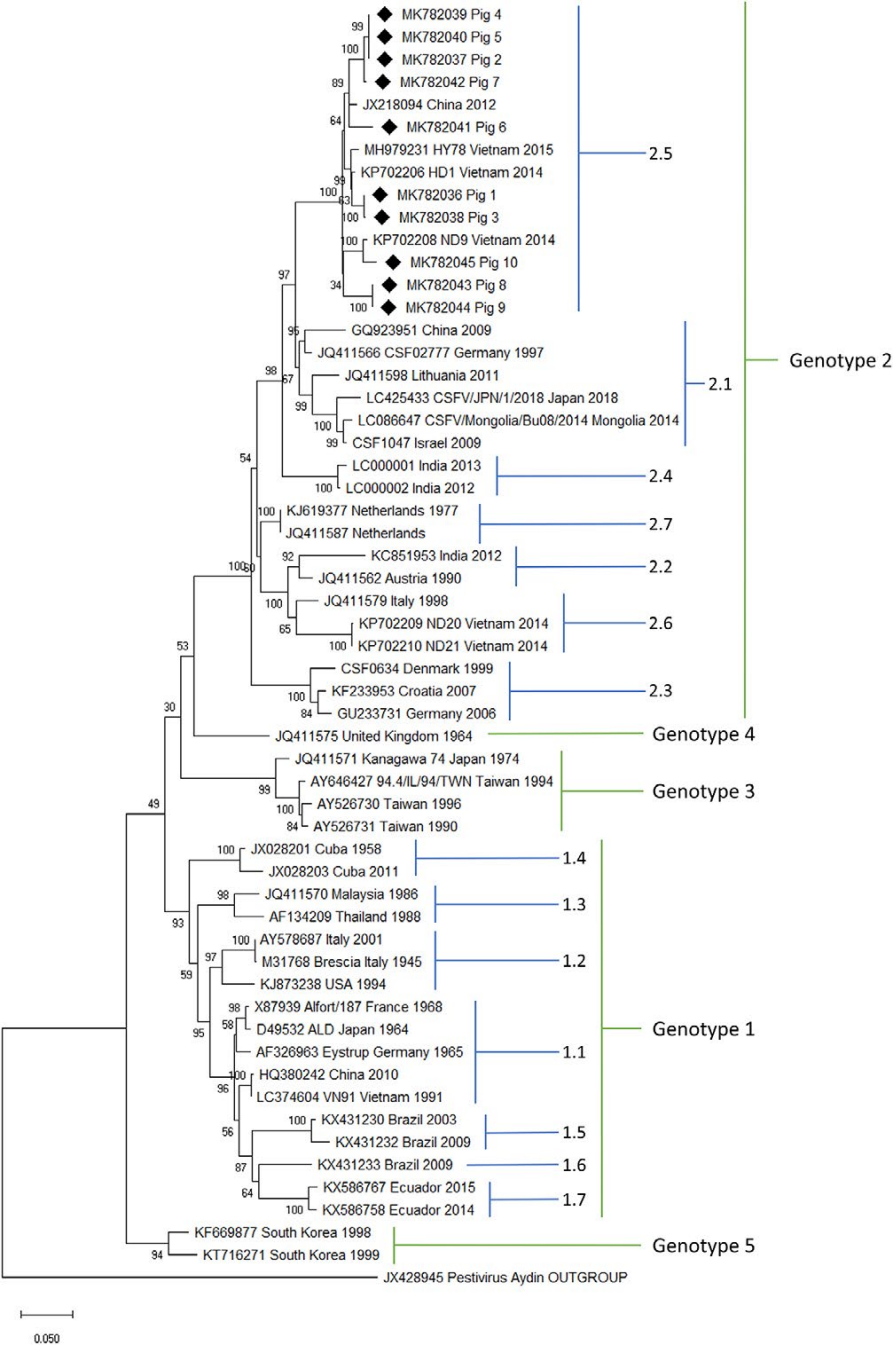
Phylogenetic tree for the full classification system of classical swine fever isolates based on 1,119 nucleotide sequence of E2 region. The isolates in this study are indicated by the diamond symbol. ML, maximum likelihood

**FIGURE 5:**
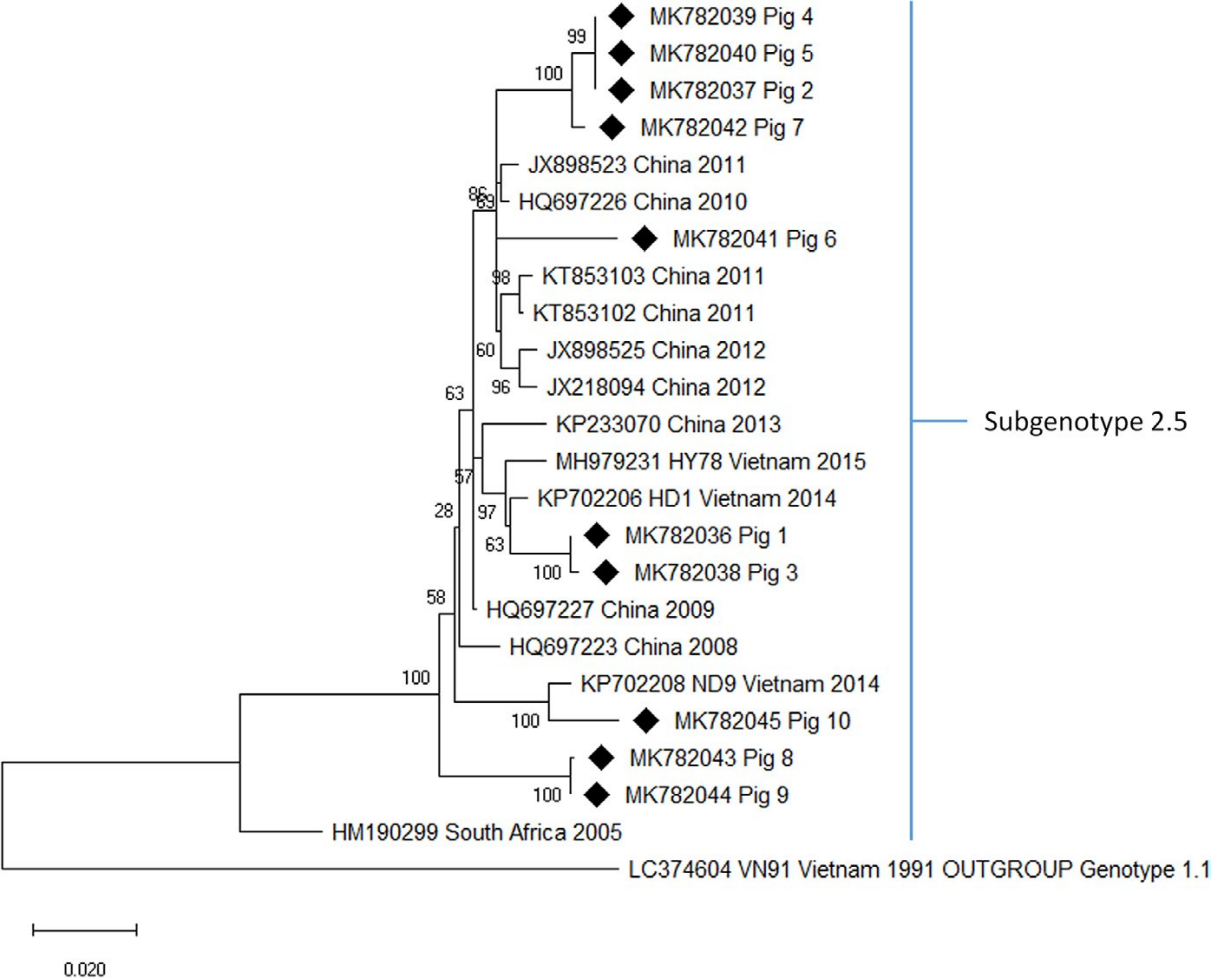
Phylogenetic tree for classical swine fever subgenotype 2.5 isolates based on 1,119 nucleotide sequence of E2 region. The isolates in this study are indicated by the diamond symbol. ML, maximum likelihood

The phylogenetic tree in Figure 4 shows the CSFV isolates according to the full classification system used in this study. CSFV in all 10 pigs belonged to subgenotype 2.5. All isolates shared 94%–100% sequence identity. Figure 5 shows the phylogenetic relationship of subgenotype 2.5. The CSFV strain in Pig 1 and Pig 3 was 98.3% and 98.4% identical, respectively, to the HD1 strain that circulated in Vietnam in 2014 (KP702206). In Pigs 2, 4 and 5, the CSFV was an identical strain, and it shared 97.8% sequence identity with the strains that circulated in China in 2010 (HQ697226) and 2011 (JX898523). The strain in Pig 7 was 99.2% identical to the strain in Pigs 2, 4 and 5. The strain in Pig 6 was 97.4% identical to the strain in China in 2010 (HQ697226). Pigs 8 and 9 were 96.8 and 96.7% identical, respectively, to a strain reported in China in 2009 (HQ697227). Pig 10 was 98.2% identical to the ND9 strain reported in Vietnam in 2014 (KP702208). The sequence identity matrix data are shown in Supplementary data 5.

## 4 DISCUSSION

The geographical locations of the five outbreaks described in this study are shown in Supplementary data 6. The affected herd at a farm located in Hung Yen province (Pig 1–5) showed clinical signs of conjunctivitis, as well as diarrhoea with variable states of faeces, shortly after weaning and did not respond to antibiotic treatment. These clinical signs, even though not specific, have been reported for the acute form of CSF (Blome et al., 2017). The mortality rate was 50%–53% at three to seven days after the onset of the clinical signs. In Thai Binh province, pigs weighing 80–90 kg in two separate wards showed less specific clinical signs, including appetite loss, fever and pinpoint haemorrhages on the skin for a period of one to two weeks prior to necropsy (Pigs 9 and 10). Only a few animals died (<1%) after showing clinical signs, and morbidity ranged from 10% to 40%–50% in the two affected wards. Detailed information about the outbreak situation related to Pigs 6, 7 and 8 was not available because these cases were retrieved from several sources; however, they were suspected of having subacute to chronic CSF based on the presence of ulcerative lesions in the gastrointestinal tracts and on the low mortality rate at the affected farms. In acute CSF infection by a high‐virulence strain, up to a 100% mortality rate may occur regardless of age group (Blome et al., 2017). Reportedly, piglets up to 12 weeks of age displayed the acute form the most frequently, while infection by the same CSFV strain in fattening and breeding animals produced less specific clinical signs and possible recovery (Moennig et al., 2003). Similarly, the CSF outbreak in the younger pigs caused a higher mortality rate compared to the older pigs in the different pig farms in this study.

We ruled out bacterial infections, including *Salmonella, Streptococcus suis, Actinobacillus pleuropneumonia* and *Haemophilus parasuis,* following a negative PCR detection of those pathogens. In addition, PCR detection of viral pathogens, including porcine reproductive and respiratory syndrome virus and swine influenza virus, were negative in all 10 pigs. However, PCV2 and PCMV antigen was detected by PCR in a few of the cases. The characteristic histopathological lesions caused by PCV2 or PCMV infection were lacking in all of the 10 cases studied; this included the absence of granulomatous inflammation in lymphoid tissue and intracytoplasmic inclusions caused by PCV2 or intranuclear cytomegalovirus inclusions. Thus, we suspected that these were subclinical infections.

The histopathological findings showed similarities as well as marked differences in the weaner pigs (Pigs 1–5) when compared to the older animals (Pigs 6–10). Assigning a CSF form to different age groups was challenging in this study because the histomorphological lesions differed appreciably from those described in the literature. None of the pigs studied showed pathognomonic splenic infarction or any distinctive vascular changes, such as endothelial necrosis or fibrinoid necrosis of the arterial wall of the lymphoid tissues, which are classic findings in CSF (Robinson & Robinson, 2016). In this study, CSF lesions, including lymphoid depletion in the tonsil, lymph nodes and spleen and histiocytic hyperplasia in the spleen, were consistent in all cases; however, a more severe necrosis in the lymph nodes tended to be more common in the older pigs than in the weaner pigs. A longer duration of disease period or clinical signs seen in those pigs before death may have contributed to these findings.

In chronic CSF, findings such as colonic button ulcers, less severe lymphoid depletion and lack of haemorrhagic lesions have been reported (Blome et al., 2017; Chander, Nandi, Ravishankar, Upmanyu, & Verma, 2014; Moennig et al., 2003). Splenic extramedullary haematopoiesis was described in pigs chronically infected with the low‐virulence CSF Kanagawa strain at 30 days post‐inoculation (Narita et al., 2000). A similar finding of extramedullary haematopoiesis in the spleen tissue was seen in Pig 10, in addition to gastrointestinal ulcers. Contrary to the findings described in chronic CSF, we did not observe a lesser severity of haemorrhagic lesions and lymphoid depletion when those lesions in older pigs were compared to the weaner pigs that showed acute CSF in this study. The severity of interstitial nephritis and interstitial pneumonia was also increased in the older pigs and correlated with a more chronic course of infection.

The weaner pigs in this study showed marked haemorrhagic lesions, as reported in acute CSF, but they lacked the generalized vasculitis and lymphoid necrosis described in acute CSF caused by the high‐virulence CSFV strains (Narita et al., 2000; Belák et al., 2008). Thus, we suspected that our strain was a moderately virulent CSFV strain that requires a longer period of infection to cause the more characteristic lesions in the host. In addition, secondary infections, which are more common in the subacute to chronic stage of CSF, were seen in Pigs 5 and 6, which showed necrotizing gastroenteritis complicated by a *Balantidium* organism. Nevertheless, the histological characteristics of a subacute CSF were not easy to specify because of the lack of clinical definitions, as well as histopathological assessment, in the literature.

Until recently, CSFV was divided into three genotypes and three or four subgenotypes (Blome et al., 2017). A new scheme for genotyping CSFV was proposed with the addition of new genotypes (4 and 5) and seven subgenotypes for genotypes 1 and 2 (Rios et al., 2018). This classification has not yet been entered into the main classification system, but we strongly recommend its utilization to harmonize the CSF classification system to improve the analysis of CSF. Following the new genotyping scheme, all naturally infected pigs in this study were classified into subgenotype 2.5. The CSFV strains circulating in Vietnam during the 2014 outbreak were reported as subgenotype 2.1 (Hung et al., 2017) and then re‐classified as subgenotype 2.5 and 2.6 (Rios et al., 2018). In the same region, VN91, a CSFV strain isolated in Hung Yen province in 1991, belongs to subgenotype 1.1, which is similar to the genotype strain commonly used in the CSF vaccine in Vietnam (Kamakawa, Thu, & Yamada, 2006; Tran, Dang, Nguyen, Miyazawa, & Kokuho, 2018). A gap of almost three decades existed between reports of the strain in 1991 and the strains detected in 2014, as well as in this study. During this gap, a shift occurred in genotype 2 over the historical genotype 1 in Vietnam.

Despite the emergence of new genotypes of CSFV, its genetic diversity has not resulted in serotypes or negative effects on vaccine efficacy because the virus is highly stable (Vanderhallen, Mittelholzer, Hofmann, & Koenen, 1999). In this study, the pig farms in Quang Nihn and Thai Binh provinces had implemented CSF vaccinations in sows and piglets, and yet the herd had contracted the disease. Vaccination was unable to induce protective immunity against the circulating strains. Therefore, other factors, such as a poorly timed implementation of vaccines, should be seriously considered by the veterinarians in these provinces.

Understanding the pathological changes and their relationship to the virulence of CSFV will require further pathological descriptions, accompanied by genetic data, collected by researchers worldwide in future studies. With a few exceptions, such as the low‐virulence strain CSF0911 Glentorf in genotype 1.1 and the high‐virulence strain CSF1047 Israel in genotype 2.1, the most virulent strains are found in genotype 1 and the moderately virulent strains in genotype 2 (Beer et al., 2015; Lohse et al., 2012). In this study, we included the phylogenetic tree analysis of the CSFV strain with the available E2 sequences and their reported virulence using the new classification system (Figure 4). Most pathological studies conducted in the early 2000s employed the highly virulent CSFV strains, including Eystrup (AF326963), Alfort/187 (X87939), ALD (D49532) and Brescia (M31768), which belong to subgenotypes 1.1 and 1.2. In 2009, subgenotype 1.5 Tiangua strain (KX431230) and subgenotype 1.6 Macapa strain (KX431233), which were isolated in Brazil, appeared as acute forms of CSF with high mortality (Silva et al., 2017). In this study, our CSF cases did not show the severe histopathological changes that were related to the necrosis and generalized vasculitis associated with the high‐virulence Alfort/187 or ALD strains.

The majority of CSFV reported for genotype 2 in the phylogenetic tree show moderate virulence (Enkhbold et al., 2017; Floegel‐Niesmann et al., 2003; Lohse et al., 2012; Postel et al., 2019). In cases of moderately virulent CSFV, IHC detected the viral antigen prior to the appearance of the histopathological lesions (Belák et al., 2008). Similarly, in this study, a substantial amount of CSFV positive antigen was seen even in the absence of necrotizing lesions in the lymph node tissues of the weaned pigs. In both age groups, no difference was noted in the antigen distributions or specific tissue tropisms. The lymphoid organ was the main target tissue for CSFV replication, before the antigen distributed among non‐lymphoid tissues, including endothelium, epithelium and nervous tissues (de las Mulas et al., 1997).

Experimental CSF infection by the highly virulent Alfort 187 strain did not lead to CSFV in the neuron, according to the IHC findings (Gómez‐Villamandos et al., 2006). In addition, the CSFV‐infected neurons may have little involvement in CSF pathogenesis, as neurological signs were also produced solely by non‐suppurative meningoencephalitis lesions (Gómez‐Villamandos et al., 2006). In this study, in all cases, neurotropism was evident in the small neurons of the brain and/or in the ganglia of myenteric plexus, but with no clear histological changes. According to the field veterinarian observations, neurological signs were seen during the outbreak in this study, but we could not identify a specific case among the studied cases.

Genotypes 3, 4 and 5 were previously grouped into genotype 3, and variable forms of CSF were reported. In the new classification system, the genotype 3 strain in Taiwan (AY646427) was reported to be moderately virulent and pathologically similar to the low‐virulence strain Kanagawa 74 (JQ411571) in Japan (Lin et al., 2007; Narita et al., 2000). No information is available regarding the virulence of the strain isolated in the United Kingdom, which belongs to genotype 4. In the genotype 5 strains in South Korea, YI9908 (KT716271) showed acute CSF, while JJ9811 (KF669877) showed milder clinical signs of CSF in experimental conditions (Lim et al., 2016). The lack of information, especially about genotypes 4 and 5, necessitates further data collection and analysis.

Following the outbreak of African swine fever (ASF) in February 2019, three million pigs were culled in Vietnam, as reported on 4 July 2019 (The Food and Agriculture Organisation of the United Nations, 2019). This situation will have drastic effects on the epidemiology of CSF in the affected region. ASF, as another haemorrhagic viral disease, needs to be differentiated from CSF by laboratory tests (Chander et al., 2014). In this study, we ruled out ASF infection by the negative IHC result using the rabbit polyclonal ASFV phosphoprotein p30 antibody (Alpha Diagnostic International) in various tissues (data not shown).

This study characterized the histopathological changes in domestic pigs that contracted an infection by the moderately virulent subgenotype 2.5 CSFV circulating in northern Vietnam in early 2018. Therefore, the results of this study may contribute to future prospective studies on the disease in those affected regions.

## CONFLICT OF INTEREST

The authors declare that they have no conflicts of interest.

## AUTHOR CONTRIBUTION

Uda Zahli Izzati: Data curation; Formal analysis; Investigation; Methodology; Software; Visualization; Writing‐original draft; Writing‐review & editing. Nguyen Thi Hoa: Conceptualization; Data curation; Investigation; Methodology; Resources. Nguyen Thi Lan: Conceptualization; Formal analysis; Funding acquisition; Project administration; Resources. Nguyen Van Diep: Data curation; Resources; Writing‐review & editing. Naoyuki Fuke: Formal analysis; Investigation; Methodology; Writing‐review & editing. Takuya Hirai: Formal analysis; Supervision; Validation. Ryoji Yamaguchi: Conceptualization; Data curation; Formal analysis; Funding acquisition; Investigation; Methodology; Project administration; Resources; Supervision; Validation; Visualization; Writing‐review & editing.

### PEER REVIEW

The peer review history for this article is available at https://publons.com/publon/10.1002/vms3.339.

## Supporting information

Sup data S1

Sup data S2

Sup data S3

Sup data S4

Sup data S5

Sup data S6
