# Supplementary material for: Pathology of the outbreak of subgenotype 2.5 classical swine fever virus in northern Vietnam
Source: Vet Med Sci. 2020 Aug 11;7(1):164–74. doi: 10.1002/vms3.339 (PMC7840204; doi:10.1002/vms3.339)
Supplement: Supplementary file 1 — Sup data S1 [file VMS3-7-164-s001.pdf]

Supplementary data 1. Primers used for detection of various viral and bacterial pathogens in CSFV infected pigs, Vietnam, 2018.

No	Pathogens	Primer	Expected band size/value
1	**Porcine reproductive and respiratory syndrome virus**<br>ORF7 gene<br>Guarino et al., 1999	PRRS F: GCTGTTAAACAGGGAGTGG<br>PRRS R: CGCCCTAATTGAATAGGTGAC	508bp
2	**Porcine circovirus type 2**<br>ORF1 gene<br>Yang et al., 2003	PCV2F1: GAAGAATGGAAGAAGCGG<br>PCV2R1: CTCACAGCAGTAGACAGGT	360bp
3	**Porcine cytomegalovirus**<br>gB gene<br>Hamel et al., 1999	PCMVF:<br>CCCTGATCTTAAATGACGAGGACGTGAC<br>PCMVR:<br>ACCGTCTGAGAGAGACTGAACTTCTCTGACAC	413bp
4	***Streptococcus suis***<br>gdh gene<br>Okwumabua et al., 2002	StS-F: GCAGCGTATTCTGTCAAACG<br>StS-R: CCATGGACAGATAAAGATGG	688bp
5	***Actinobacillus pleuropneumoniae***<br>apxIV gene<br>Xiao et al., 2006	AP-IVF: ATACGGTTAATGGCGGTAATGG<br>AP-IVR: ACCTGAGTGCTCACCAACG	346bp
6	***Haemophilus parasuis***<br>16S small subunit ribosomal RNA gene<br>Oliveira et al., 2001	HPf: GTGATGAGGAAGGGTGGTGT<br>HPr: GGCTTCGTCACCCTCTGT	821bp
7	***Salmonella***<br>invA gene<br>Rahn et al., 1992	Sal.F set3:<br>GTGAAATTATCGCCACGTTCGGGCAA<br>Sal.R set3: TCATCGCACCGTCAAAGGAACC	284bp
8	**Influenza A virus of swine**<br>M gene<br>OIE, 2018	M25f: AGATGAGTCTTCTAACCGAGGTCG<br>M64 probe: FAM-<br>TCAGGCCCCCTCAAAGCCGA-BHQ<br>M124R: TGCAAAAACATCTTCAAGTCTCTG<br>M124-SIVR: TGCAAAGACACTTTCCAGTCTCTG	Ct ≤35

References:

1. Guarino, H., Goyal, S. M., Murtaugh, M. P., Morrison, R. B., & Kapur, V. (1999). Detection of Porcine Reproductive and Respiratory Syndrome Virus by Reverse Transcription-Polymerase Chain Reaction using Different Regions of the Viral Genome. Journal of Veterinary Diagnostic Investigation, 11, 27–33. https://doi.org/10.1177/104063879901100104
2. Yang, J. S., Song, D. S., Kim, S. Y., Lyoo, K. S., & Park, B. K. (2003). Detection of Porcine Circovirus Type 2 in Feces of Pigs with or without Enteric Disease by Polymerase Chain Reaction. Journal of Veterinary Diagnostic Investigation, 15, 369–373. https://doi.org/10.1177/104063870301500412
3. Hamel, A. L., Lin, L., Sachvie, C., Grudeski, E., & Nayar, G. P. S. (1999). PCR Assay for Detecting Porcine Cytomegalovirus. Journal of Clinical Microbiology, 37, 3767–3768. https://doi.org/10.1128/JCM.37.11.3767-3768.1999
4. Okwumabua, O., O'Connor, M., & Shull, E. (2003). A polymerase chain reaction (PCR) assay specific for Streptococcus suis based on the gene encoding the glutamate dehydrogenase. FEMS Microbiology Letters, 79-84. https://doi.org/10.1111/j.1574-6968.2003.tb11501.x
5. Xiao, G.S., Cao S.J., Duan, L.L., Wen, X.T., Ma, X.P., Chen, H.M. (2006). Identification and detection of Actinobacillus pleuropneumoniae in infected and subclinically infected pigs by multiplex PCR based on the genes ApxIVA and OmlA. Agricultural Sciences in China, 5, 146–154. https://doi.org/10.1016/S1671-2927(06)60032-1
6. Oliveira, S., Galina, L., & Pijoan, C. (2001). Development of a PCR Test to Diagnose Haemophilus Parasuis Infections. Journal of Veterinary Diagnostic Investigation, 13, 495–501. https://doi.org/10.1177/104063870101300607
7. Rahn, K., De Grandis, S. A., Clarke, R. C., McEwen, S. A., Galán, J. E., Ginocchio, C., Curtiss, R., & Gyles, C. L. (1992). Amplification of an invA gene sequence of Salmonella typhimurium by polymerase chain reaction as a specific method of detection of Salmonella. Molecular and Cellular Probes, 6, 271–279. https://doi.org/10.1016/0890-8508(92)90002-F
