# Supplementary material for: Pathology of the outbreak of subgenotype 2.5 classical swine fever virus in northern Vietnam
Source: Vet Med Sci. 2020 Aug 11;7(1):164–74. doi: 10.1002/vms3.339 (PMC7840204; doi:10.1002/vms3.339)
Supplement: Supplementary file 3 — Sup data S3 [file VMS3-7-164-s003.pdf]

Supplementary data 3: PCR detection of various viral and bacterial pathogens from the tonsil/spleen homogenate in pigs naturally infected with CSF in northern Vietnam, 2018

Pig number	PCV2	PRRSV	SIV	PCMV	*Salmonella*	*Strep. suis*	APP	*H. parasuis*
1	+	-	-	-	-	-	-	-
2	+	-	-	-	-	-	-	-
3	+	-	-	-	-	-	-	-
4	-	-	-	-	-	-	-	-
5	+	-	-	-	-	-	-	-
6	-	-	-	+	-	-	-	-
7	-	-	-	+	-	-	-	-
8	-	-	-	-	-	-	-	-
9	-	-	-	+	-	-	-	-
10	-	-	-	+	-	-	-	-

PCV2: porcine circovirus 2; PRRSV: porcine reproductive and respiratory syndrome virus; SIV: swine influenza virus (influenza A); PCMV: Porcine cytomegalovirus; *Strep. suis*: *Streptococcus suis*; APP: *Actinobacillus pleuropneumoniae*; *H. parasuis*: *Haemophilus parasuis*; -: negative; +: positive
