# Supplementary material for: Pathology of the outbreak of subgenotype 2.5 classical swine fever virus in northern Vietnam
Source: Vet Med Sci. 2020 Aug 11;7(1):164–74. doi: 10.1002/vms3.339 (PMC7840204; doi:10.1002/vms3.339)
Supplement: Supplementary file 4 — Sup data S4 [file VMS3-7-164-s004.pdf]

Supplementary data 4. Gross lesions of classical swine fever in northern Vietnam, 2018

Organ	Gross lesions	Acute to subacute cases					Subacute to chronic cases				
		Pig number									
		1	2	3	4	5	6	7	8	9	10
Lympho-reticular organ	Dark discoloration of spleen	+	-	-	+	-	-	+	+	-	-
	Sinus hemorrhage in lymph nodes	+	+	-	+	+	-	+	+	-	+
	Tonsillar necrosis	+	-	-	-	-	-	-	nd	-	-
Skin	Petechiation and earlobes erythema	+	-	+	+	+	-	+	-	+	-
Larynx	Petechiation	-	+	-	+	+	-	+	-	-	-
Gastro-intestinal tract	Ulcer (stomach)	-	-	-	-	-	+	+	-	+	+
	Serosal petechiation (stomach)	-	-	-	+	-	-	+	-	-	-
	Mucosal petechiation (small intestines)	-	-	-	+	+	-	-	-	-	-
	Button ulcers (colon)	-	-	-	-	+	+	-	-	-	+
Lung	Petechial to blotchy hemorrhages of pleural surface	+	-	+	-	+	-	+	-	-	-
	Generalized congestion	+	-	-	-	+	+	-	+	+	+
Kidney	Petechiation	+	+	-	+	-	-	-	+	-	+
	Generalized congestion	+	-	-	-	-	-	+	+	-	+
Urinary bladder	Mucosal petechiation	+	-	+	-	+	-	-	-	+	-
Heart	Pericardial petechiation	-	-	-	-	+	-	+	+	-	-
Brain	Meningeal congestion/ hemorrhages	+	-	-	+	+	-	nd	nd	nd	nd
