# Supplementary material for: Pathology of the outbreak of subgenotype 2.5 classical swine fever virus in northern Vietnam
Source: Vet Med Sci. 2020 Aug 11;7(1):164–74. doi: 10.1002/vms3.339 (PMC7840204; doi:10.1002/vms3.339)
Supplement: Supplementary file 6 — Sup data S6 [file VMS3-7-164-s006.pdf]

Supplementary data 6: Geographical location of pig farms affected in the classical swine fever outbreaks in northern Vietnam, 2018

Provinces

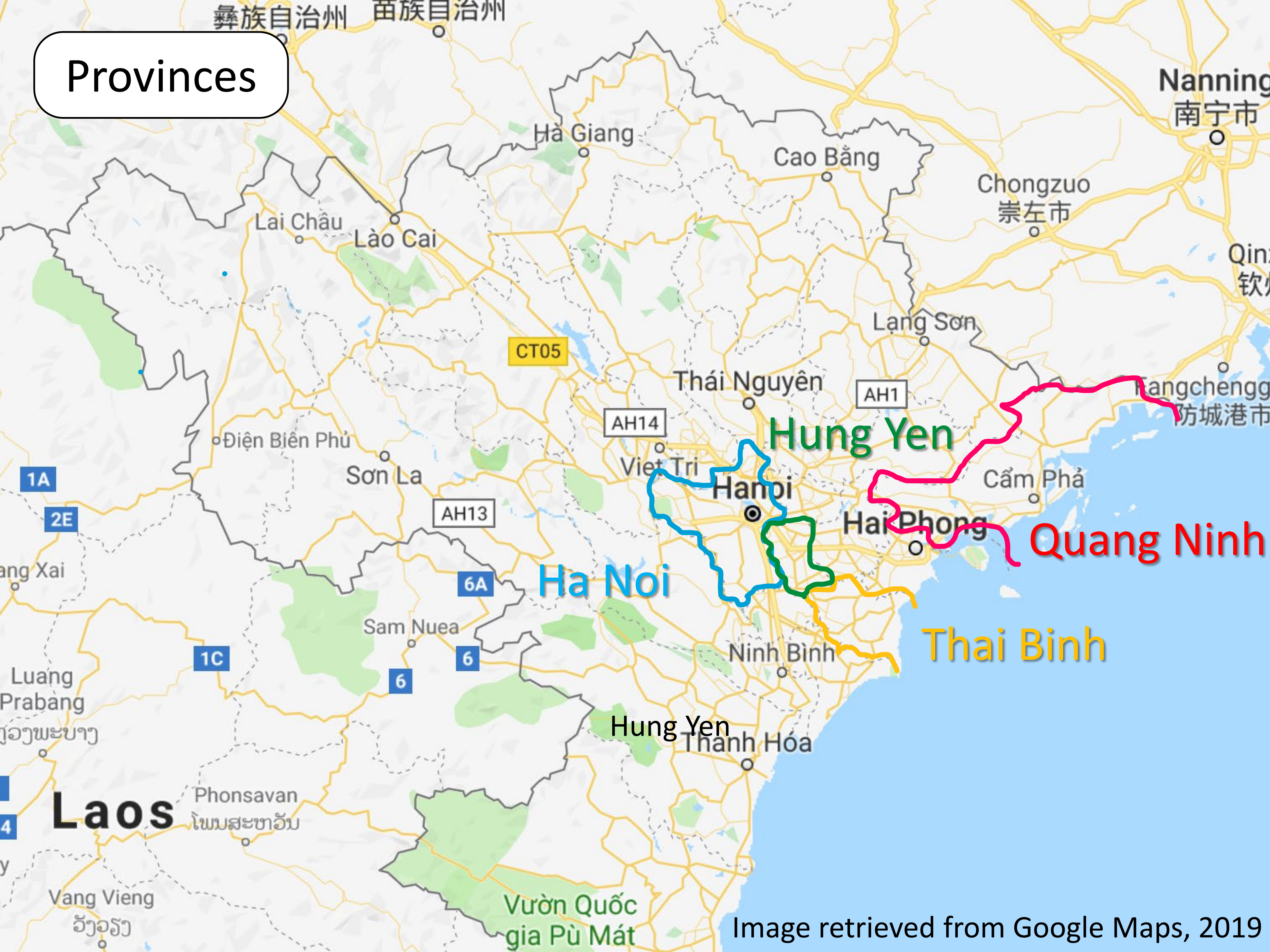

Image retrieved from Google Maps, 2019

Districts

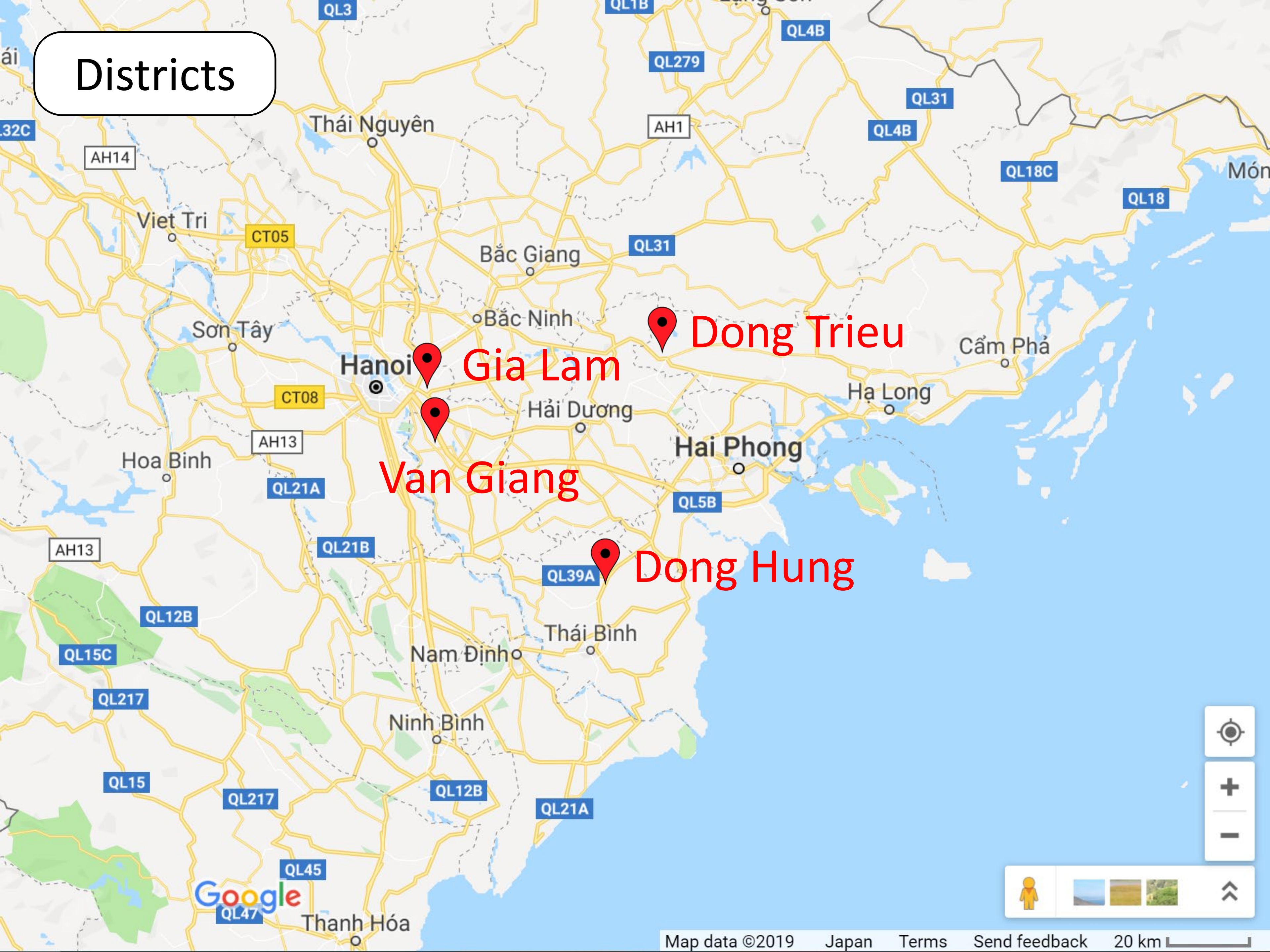
Gia Lam
Van Giang
Dong Trieu
Dong Hung
Thái Nguyên
Viet Tri
Bắc Giang
Bắc Ninh
Sơn Tây
Hanoi
Hải Dương
Hai Phong
Cẩm Phả
Hạ Long
Hoa Binh
Thái Bình
Nam Định
Ninh Bình
Thanh Hóa
QL3
QL1B
QL4B
QL279
QL31
AH1
QL18C
QL18
AH14
CT05
CT08
AH13
QL21A
QL5B
QL21B
QL39A
QL12B
QL15C
QL217
QL15
QL45
QL47
Google
Map data ©2019
Japan
Terms
Send feedback
20 km
